# A MYST family histone acetyltransferase, MoSAS3, is required for development and pathogenicity in the rice blast fungus

**DOI:** 10.1111/mpp.12856

**Published:** 2019-07-30

**Authors:** Akanksha Dubey, Jongjune Lee, Seomun Kwon, Yong‐Hwan Lee, Junhyun Jeon

**Affiliations:** ^1^ Department of Biotechnology, College of Life and Applied Sciences Yeungnam University Gyeongsan Gyeongbuk 38541 Korea; ^2^ Department of Agricultural Biotechnology, College of Agriculture and Life Sciences Seoul National University Seoul 08826 Korea; ^3^ Center for Fungal Genetic Resources Seoul National University Seoul 08826 Korea; ^4^ Plant Immunity Research Center Seoul National University Seoul 08826 Korea; ^5^ Present address: Heinrich‐Heine University Düsseldorf, Institute for Microbiology, Cluster of Excellence on Plant Sciences Düsseldorf 40204 Germany

**Keywords:** development, histone acetyltransferase, MoSAS3, pathogenicity, rice blast

## Abstract

Histone acetylation has been established as a principal epigenetic regulatory mechanism in eukaryotes. Sas3, a histone acetyltransferase belonging to the largest family of acetyltransferase, MYST, is the catalytic subunit of a conserved histone acetyltransferase complex. To date, the functions of *Sas3* and its orthologues have been extensively studied in yeast, humans and flies in relation to global acetylation and transcriptional regulation. However, its precise impact on development and pathogenicity in fungal plant pathogens has yet to be elucidated. Considering the importance of *Sas3* in H3K14 acetylation, here we investigate the roles of its orthologue in the rice blast fungus, *Magnaporthe oryzae* (*Pyricularia oryzae*). Unlike a previously reported *Sas3* deletion in yeast, which led to no remarkable phenotypic changes, we found that *MoSAS3* deletion alone had a profound effect on fungal growth and development, including asexual reproduction, germination and appressorium formation in *M. oryzae*. Such defects in pre‐penetration development resulted in complete loss of pathogenicity in the deletion mutant. Furthermore, genetic analysis of *MoSAS3* and *MoGCN5* encoding a Gcn5‐related *N*‐acetyltransferase family histone acetyltransferase suggested that two conserved components of histone acetylation are integrated differently into epigenetic regulatory mechanisms in the yeast and a filamentous fungus. RNA‐seq analysis of Δ*Mosas3* showed two general trends: many DNA repair and DNA damage response genes are up‐regulated, while carbon and nitrogen metabolism genes are down‐regulated in Δ*Mosas3*. Our work demonstrates the importance of MYST family histone acetyltransferase as a developmental regulator and illuminates a degree of functional variation in conserved catalytic subunits among different fungal species.

## Introduction

Histone modification is a conserved post‐translational modification (PTM) that plays a pivotal role in the epigenetic regulation of gene expression and chromatin in eukaryotes (Kouzarides, 2007). Modification of histones is catalysed by specialized histone‐modifying enzymes (HMEs) such as histone acetyltransferases/deacetylase (HATs/HDACs) and histone methyltransferases/demethylases (HMTs/HDMs) that function via addition or removal of acetyl and methyl residues from the histone proteins, respectively. Since the discovery of histone acetylation in 1964 (Allfrey and Mirsky, 1964; Allfrey *et al.*, 1964), the antagonistic interplay of HATs and HDACs has been established as one of the principal epigenetic regulatory mechanisms.

HATs are one of the best‐characterized groups of HMEs, which use acetyl‐CoA as a substrate for the acetylation of lysine (K) residues within both the tail and globular domains of histones to poise the gene for active transcription (Dubey and Jeon 2017; Jeon *et al.*, 2014; Sterner and Berger, 2000). However, as an increasing number of non‐histone targets of previously known HATs and HDACs are being discovered, a new classification placed these enzymes under the umbrella of lysine acetyltransferase (KATs) and deacetylases (KDACs) (Allis *et al.*, 2007). Ranging from yeast to humans, several HATs have been identified that can be classified, based on the presence of conserved structural motifs, into five families including Gcn5‐related *N*‐acetyltransferase (GNAT) and MYST (members MOZ, MOF, MORF, Ybf2/Sas3, Sas2, Tip60 and HBO1) families (Jeon *et al.*, 2014; Sterner and Berger, 2000).

In fungi, histone acetylation/deacetylation was shown to be important for regulation of growth, asexual development, secondary metabolite production and pathogenicity (Ding *et al.*, 2010; Jeon *et al.*, 2014). Among the five families of HATs, the GNAT superfamily has been extensively studied. In particular, Gcn5 orthologues have been demonstrated to be required for asexual development in *Aspergillus nidulans*, dimorphism and virulence in *Ustilago maydis*, and regulation of autophagy in *Magnaporthe oryzae* (anamorph *Pyricularia oryzae*), respectively (Canovas *et al.*, 2014; Gonzalez‐Prieto *et al.*, 2014; Li *et al.*, 2017). In addition, several studies investigated roles of Gcn5 and Sas2 orthologues in regulation of fungal secondary metabolism (Gomez‐Rodriguez *et al.*, 2018; Zhang *et al.*, 2018). Comparatively, however, the MYST family of HATs has not gained much attention. Lysine acetyltransferase 6 (KAT6) is an important member of the MYST family of HATs that catalyses acetylation of histone substrates H3K9, K14 and K23 (Huang *et al.*, 2016). Several KAT6 homologues have been identified, including MOZ (monocytic leukemia zinc finger)/MORF (MOZ‐related factor) in humans (Huang *et al.*, 2016), Sas3 (something about silencing) in yeast (Reifsnyder *et al.*, 1996) and ENOK (enoki mushroom) in *Drosophila* (Scott *et al.*, 2001). Phylogenetic analysis showed that the homologues Sas2 and Sas3 from yeast share homology with the human MOZ oncogene (Lafon *et al.*, 2007) and other MYST members owing to the presence of a conserved domain containing an atypical zinc finger and a putative acetyl‐CoA binding motif (Takechi and Nakayama, 1999). These domains are important for the functioning of the MYST proteins. Mutant analysis of Sas3 through amino acid substitution in *Saccharomyces cerevisiae* revealed that both the acetyl‐CoA binding motif and the zinc finger motif are essential for HAT activity (Takechi and Nakayama, 1999). To date, functionally, MOZ/MORF have been implicated in leukaemogenesis (Rokudai *et al.*, 2009, 2013), Sas3 in controlling global acetylation level in yeast in association with Gcn5 (Rosaleny *et al.*, 2007), and ENOK in total global acetylation level in *Drosophila* (Feller *et al.*, 2015; Huang *et al.*, 2014). However, there is an evident lack of reports on the role of Sas3 in filamentous fungi and its impact on fungal development and pathogenicity.

In *S. cerevisiae*, Sas3, a member of the MYST family of HATs and the catalytic subunit of the conserved 400‐kDa NuA3 (nucleosomal acetyltransferase of histone H3) HAT complex, was originally discovered in a screen for mutations enhancing loss of silencing in *Sir1* (HDAC) deletion mutant background (Reifsnyder *et al.*, 1996). Sas3 of *S. cerevisiae* was shown to have *in vitro* HAT activity (Takechi and Nakayama, 1999) and specifically acetylate H3K9 and H3K14 *in vivo* (Durant and Pugh, 2006; Vicente‐Munoz *et al.*, 2014). Being the catalytic subunit of the NuA3 complex, Sas3 is necessary for HAT activity as well as the integrity of the NuA3 complex (John *et al.*, 2000). In *S. cerevisiae*, while the presence of a single H4‐specific HAT [Esa1 (essential SAS2‐related acetyltransferase)] is essential for cell viability (Clarke *et al.*, 1999; Smith *et al.*, 1998), catalytic subunits of HATs related to H3 acetylation appear to be dispensable (Georgakopoulos and Thireos, 1992) unless deleted in double mutant combinations (Howe *et al.*, 2001). With an overlapping pattern of acetylation, NuA3 has been shown to function in close relation to Gcn5 in *S. cerevisiae* (Rosaleny *et al.*, 2007). As a result, although the deletion of Sas3 alone does not elicit any phenotype, its combined deletion with Gcn5 causes complete loss of acetyltransferase activity, resulting in reduced global H3 acetylation and cell cycle arrest that is synthetically lethal (Howe *et al.*, 2001).

In this study, we investigated the role of the *Sas3* orthologue and its histone acetylation activity in the regulation of the growth, development and pathogenicity of *M. oryzae* (Couch and Kohn, 2002; Couch *et al.*, 2005; Gomez‐Rodriguez *et al.*, 2018; Grimaldi *et al.*, 2006; Zhang *et al.*, 2016), combining genetic and transcriptomic approaches. *Magnaporthe oryzae*, an ascomycete fungus, is a devastating plant pathogen known to cause rice blast disease (Dean *et al.*, 2012; Talbot, 2003). It is a notorious fungal plant pathogen that infects a wide range of hosts and poses a threat to food security and rice production worldwide (Dean *et al.*, 2012; Maciel *et al.*, 2014; Martin‐Urdiroz *et al*., 2016; Wilson and Talbot, 2009). The fungus produces aerial conidiophores bearing conidia, which attach and germinate on the host surface to initiate fungal infection. Upon germination, the conidium produces a specialized single‐celled infection structure called an appressorium that breaches the plant surface usually protected by a cuticle layer and the cell wall (Dean, 1997; Talbot, 2003; Wilson and Talbot, 2009). Enormous turgor pressure generated inside the melanized appressorium drives the penetration hyphae into host cells (Wilson and Talbot, 2009). Such penetration is followed by transition into invasive hyphae, which requires suppression of plant immunity primarily through secretion of effector proteins (Molloy, 2010; Yan and Talbot, 2016).

Here we examined the functions of MoSAS3 as histone acetyltransferase and its impact on fungal development and pathogenesis in the rice blast fungus, compared to the yeast counterpart. Furthermore, we analysed the transcriptome of the wild‐type and the deletion mutant in order to gain insight into the role of *MoSAS3*.

## Results

### Identification and domain analysis of *MoSAS3*

**Figure 1:**
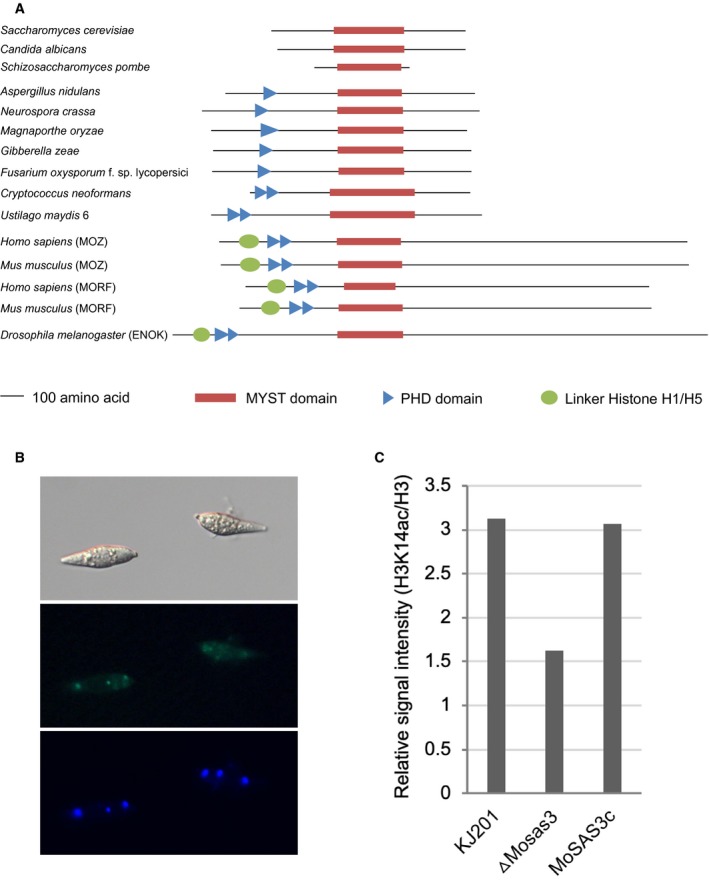
MoSAS3 as a histone acetyltransferase. (A) Domain architecture of MoSAS3 and its orthologues. (B) Localization of MoSAS3 in *Magnaporthe oryzae* spores. Green fluorescence in conidia expressing MoSAS3‐GFP (middle panel) and DAPI staining pattern (bottom panel). (C) Quantification of western blot signal for H3K14 acetylation, relative to H3. The western blot image was quantified using ImageJ software (https://imagej.nih.gov/ij/index.html).

To identify orthologues of *S. cerevisiae* Sas3, we ran a BLAST search using Sas3 sequences as a query against the NCBI nr database (Altschul *et al.*, 1990). Through the database search, we retrieved amino acid sequences of its orthologues from diverse organisms, including *M. oryzae*. A gene encoding the *Sas3* orthologue in *M. oryzae* was designated as *MoSAS3* (MGG_04615). Comparison of the domain architecture using the InterPro database (Finn *et al.*, 2017) revealed that the Sas3 orthologues possess the signature MYST‐type HAT domain in common (Fig. 1A). Close examination of MYST domains showed that the central core‐binding region to acetyl‐CoA with flanking N‐ and C‐terminal regions harbouring a C_2_H_2_ zinc‐binding domain and helix‐turn‐helix DNA‐binding motif are well conserved in all the Sas3 orthologues we examined (Lafon *et al.*, 2007; McCullough and Marmorstein, 2016; Yan *et al.*, 2000). Compared to the Sas3 of *S. cerevisiae* and *Candida albicans*, the Sas3 orthologues of filamentous ascomycetes, *Aspergillus nidulans*, *Neurospora crassa*, *M. oryzae*, *Gibberella zeae* and *Fusarium oxysporium* contain an additional PHD finger domain, which is known to be involved in recognition of H3K4me3 (Musselman and Kutateladze, 2011). Interestingly, linker histone (H1/H5) domain, which is essential for the binding of linker histone H1 or H5 to the nucleosome, was found only in human and fly.

Since Sas3 of *S. cerevisiae* is a component of NuA3 HAT complex, it was expected that MoSAS3 should be localized to the nucleus of *M. oryzae*. PSORT analysis predicted nucleus localization of MoSAS3 (95.7%) (Horton *et al.*, 2007). To test this, we made a translational fusion construct between *MoSAS3* and green fluorescence protein (eGFP) under the control of the native *MoSAS3* promoter, and introduced the construct into the wild‐type strain KJ201. Indeed, we were able to detect green fluorescence signal from the fungal nucleus under the epifluorescence microscope, although a faint/weak signal appears to be present also in the cytoplasm (Fig. 1B). Conservation of protein domains and localization strongly suggests that MoSAS3 is an evolutionarily conserved histone acetyltransferase.

### *MoSAS3* deletion impairs vegetative growth and asexual spore production

**Figure 2:**
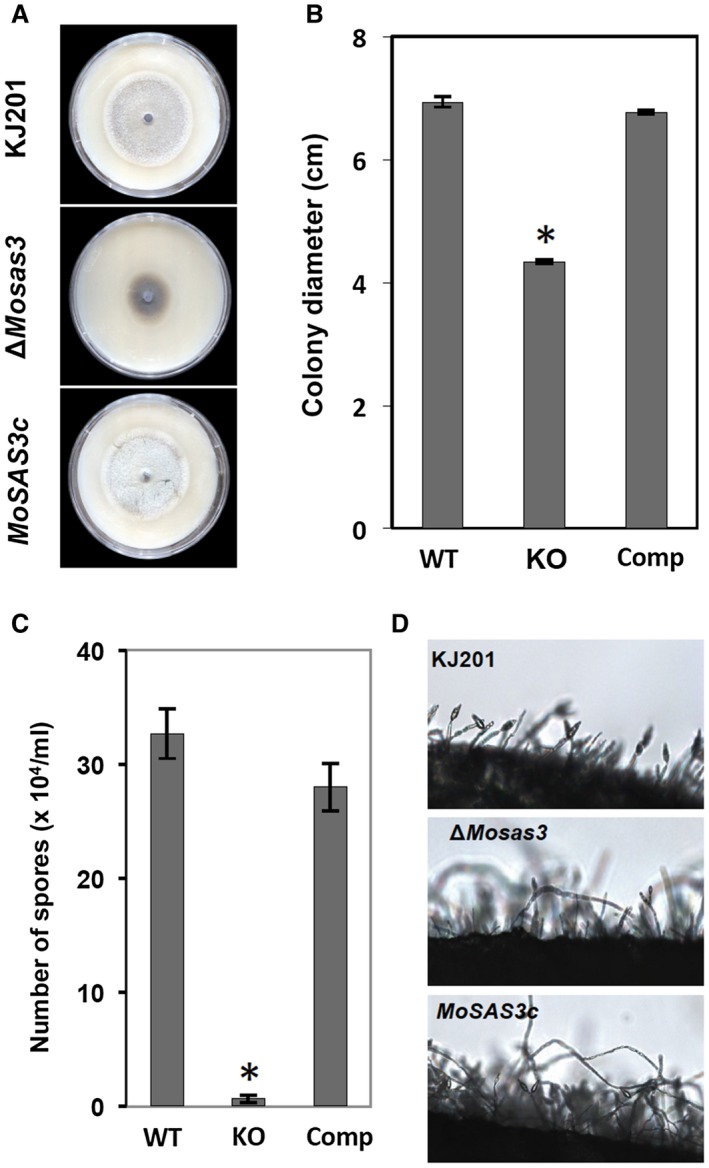
Growth and asexual reproduction of wild‐type *Magnaporthe oryzae* (KJ201, WT), Δ*Mosas3* (KO) and complementation strain (Comp, *MoSAS3c*). (A) Colony morphology and pigmentation on oatmeal agar plates. (B) Growth rate measured in colony diameter at 9 days post‐inoculation. (C) Asexual sporulation measured in the number of spores produced from individual strains. (D) Conidiophore development. Mycelial mat was scraped off and then a small agar block (1 × 1 cm) was excised and incubated for 8 h under constant fluorescence light to induce sporulation before being observed under a microscope. Asterisks indicate statistically significant differences (*P* < 0.001, Tukey HSD test).

To investigate the functions of *MoSAS3* in the development and pathogenicity of *M. oryzae*, we generated a gene deletion mutant using double‐joint PCR and protoplast transformation (Materials and Methods, and Fig. S1, see Supporting Information) and compared it to the wild‐type strain KJ201 for changes in histone acetyltransferase activity and phenotypes. Our western blot analysis using antibody raised against acetylated H3K14 showed that deletion of *MoSAS3* gene results in about 48% reduction in H3K14ac level (Figs 1C and S2A, see Supporting Information). Quantification of H3K14ac through an independent method consistently showed over 40% decreases upon deletion of *MoSAS3* (Fig. S2B, see Supporting Information), lending support to the role of MoSAS3 as a histone acetyltransferase. When the mutant was grown on oatmeal agar plates, it exhibited highly diminished growth (*c*.60% of the wild‐type) (Fig. 2A,B). The mutant colonies showed not only reduced radial growth but also a decrease in aerial hyphae masses compared to the wild‐type strain. Additionally, the mutant was more pigmented than the wild‐type (Fig. 2A).

Counting the number of asexual spores from agar plates showed that the mutant is not capable of producing as many spores as the wild‐type (Fig. 2C). Examination of conidiophore development in agar plugs revealed that such dramatic reduction in asexual spore production is attributed largely to a decrease in the number of conidiophores in Δ*Mosas3*, which is consistent with a decrease in aerial hyphae masses (Fig. 2D). Introduction of the *MoSAS3* gene back into the deletion mutant (*MoSAS3c*) was able to restore the mutant phenotypes to the wild‐type level (Fig. 2), indicating that targeted deletion of *MoSAS3* is responsible for the defects in growth and asexual reproduction.

### Germination and appressorium formation of Δ*Mosas3*

**Figure 3:**
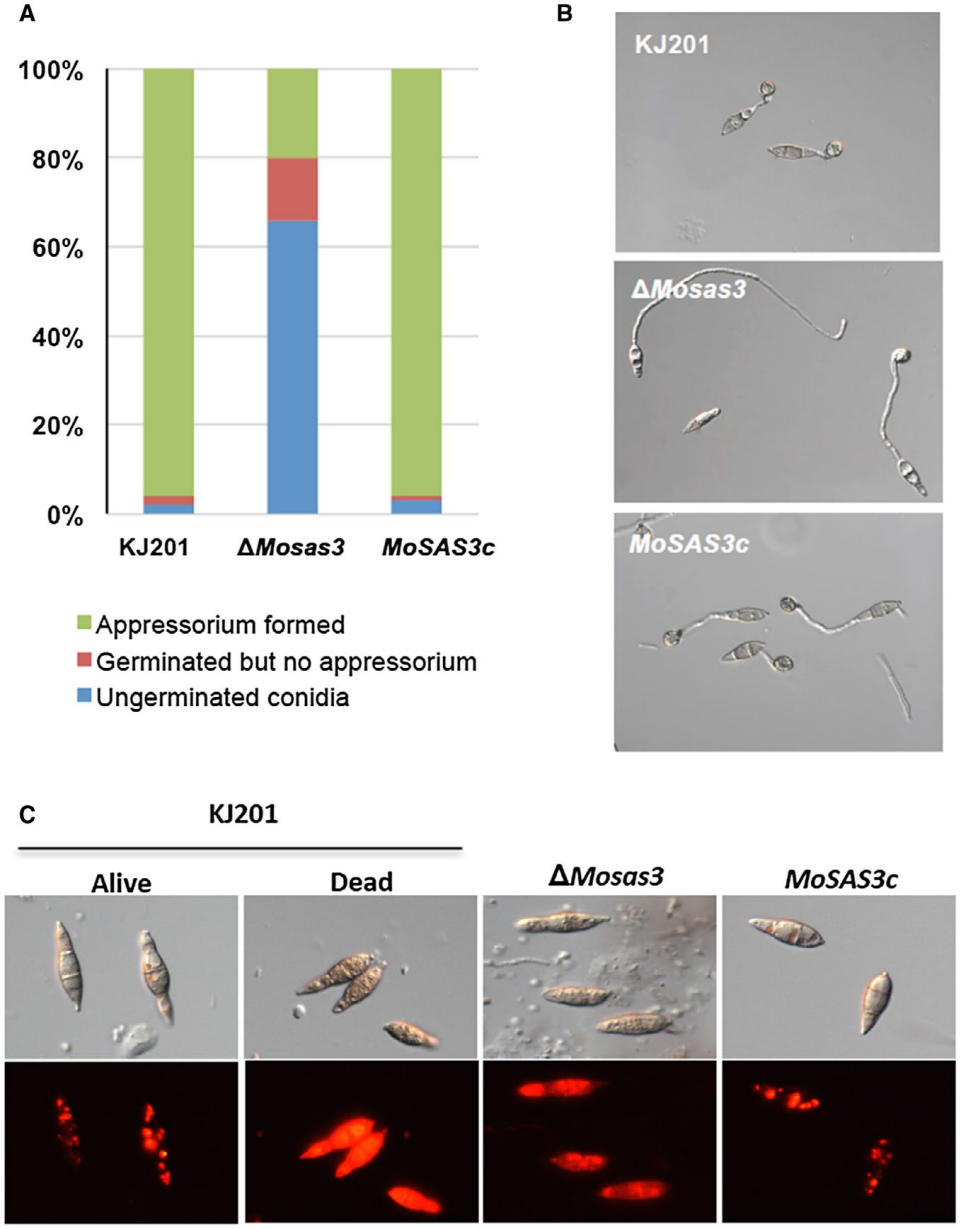
Defect in conidial germination and appressorium formation of *Magnaporthe oryzae* Δ*Mosas3*. (A) Quantitative analysis of spore germination and appressorium formation in wild‐type (KJ201), Δ*Mosas3* and complementation strain (*MoSAS3c*) after 8 h of incubation on hydrophobic surface (glass coverslip) at 25 °C. Percentages of ungerminated, germinated and appressorium‐forming conidia were counted under a light microscope. (B) Representative image for germination and appressorium formation of different strains on hydrophobic surface (plastic coverslip). (C) Spore viability test using FUN‐1 staining. Dead spores were prepared by subjecting them to microwave treatment for 1 min. Live cells localize dye to vacuoles, while dead cells display diffuse signals throughout the cytoplasm.

Next, we monitored pre‐penetration development involving germination and appressorium formation of the mutant strain compared to wild‐type. While in the wild‐type and the complementation strain, more than 95% of conidia germinated and *c*.96% of germinated conidia were able to form an appressorium*,* only 34% of the total conidia germinated, of which only 20% formed an appressorium in Δ*Mosas3* (Fig. 3A,B). Furthermore, deformed conidia were found more frequently in the deletion mutant and such deformed ones, if they germinated, tended to form a longer germ tube and an irregular appressorium (Fig. 3B).

To understand why the mutant conidia are defective in germination, we checked the viability of the mutant conidia using the FUN‐1 staining method (Essary and Marshall, 2009; Millard *et al.*, 1997). In metabolically active cells, FUN‐1 dye, which is visible as red fluorescence, concentrates at intravacuolar structures. In contrast, red fluorescence signals are diffuse in the whole cell in metabolically inactive cells. Our preliminary experiments showed that treatment of wild‐type conidia with non‐permissible temperature (65 °C) for 15 min could render the conidia inviable and completely block germination (Fig. 3C, KJ201 panels). Unlike conidia from wild‐type and complementation strains displaying punctuated fluorescence signals, the FUN‐1 staining pattern of the mutant conidia resembled that of the negative control (heat‐killed spores), suggesting that *MoSAS3* is essential for production of viable conidia.

### MoSAS3 is required for fungal pathogenicity

**Figure 4:**
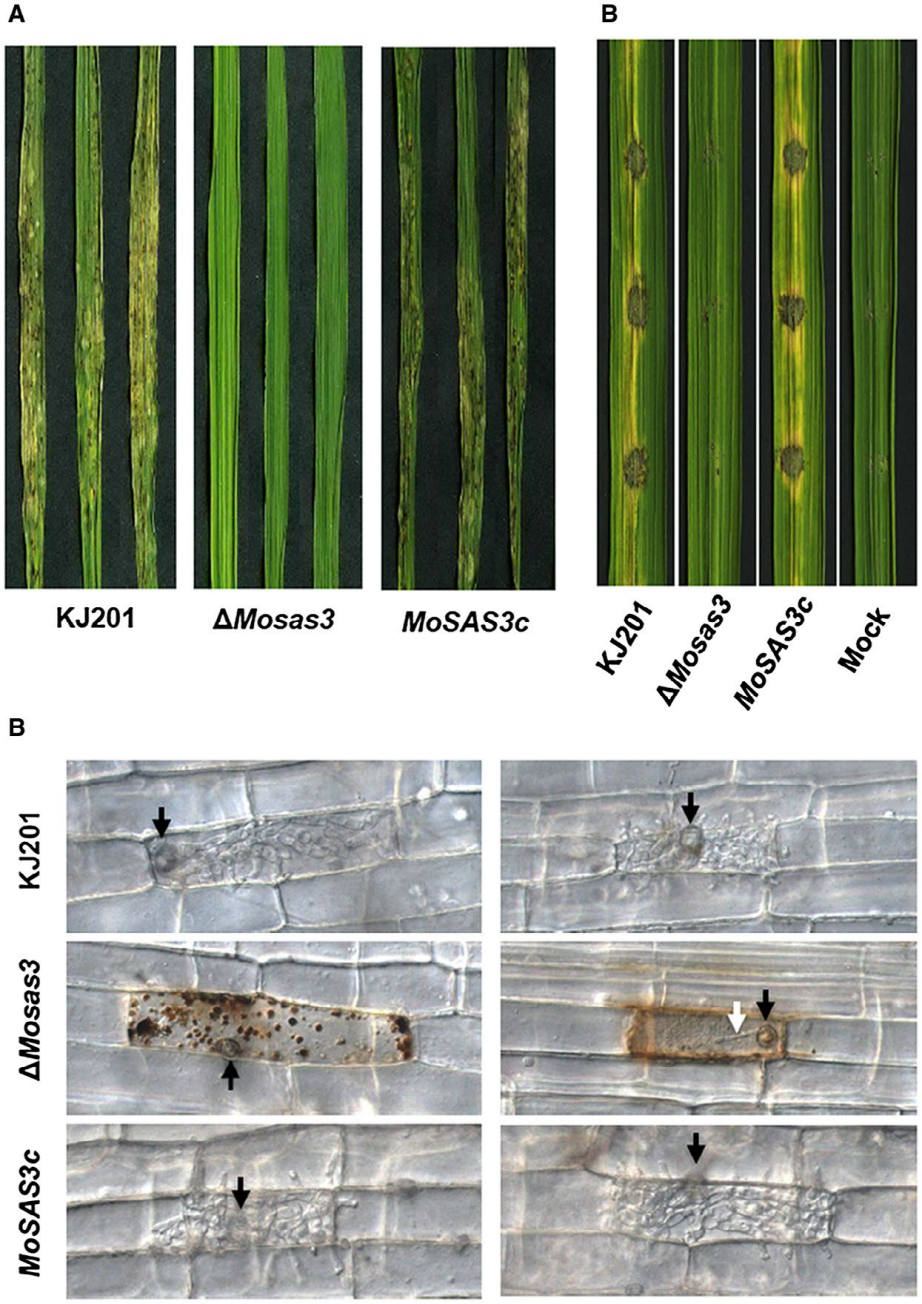
Dissection of pathogenicity defect in *MoSAS3* deletion mutant. (A) Lesion development in rice leaves 7 days post‐spray inoculation. (B) Lesion development in wound‐inoculated rice leaves 5 days post‐inoculation. Distilled water was used as a mock experiment. (C) Observation of invasive growth using rice sheath assay. Invasive growth in rice sheath cells was monitored under microscope at 48 h post‐inoculation.

In order to assess the degree to which *MoSAS3* is involved in pathogenicity, conidial suspensions prepared from plate cultures of wild‐type, Δ*Mosas3* and the complemented strain *MoSAS3c* were sprayed onto a susceptible rice cultivar, Nakdongbyeo. Disease symptom development was evaluated for inoculated plants 7 days post‐inoculation (dpi). While the wild‐type strain KJ201 and *MoSAS3c* produced typical lesions, including dark brown spots and yellowing of leaves, almost no lesions were observed on the leaves spray‐inoculated with a spore suspension of Δ*Mosas3* (Fig. 4A). It stands to reason that such loss of pathogenicity in the mutant is accountable for the observed defects in pre‐penetration development.

To determine if the mutant retains the ability to grow inside host plants, we performed wound‐inoculation by placing spore suspensions on wound sites made by pressing pipette tips against host leaves (Fig. 4B). This allows the fungus to gain direct access to the host cells without appressorium‐mediated penetration. Again, even in wound‐inoculation, the mutant was not able to cause disease symptoms, suggesting that *MoSAS3* is required for invasive growth as well. In line with these results, rice sheath assay of the mutant showed that appressoria of Δ*Mosas3* were rarely able to penetrate plant cells with poorly growing invasive hyphae. Furthermore, in many cases there was either an abundant accumulation of dark granules or discolouration of plant cells, possibly suggesting inability of the mutant to suppress the initial defence responses of host plants.

### *MoSAS3* does not genetically interact with *MoGCN5*

In *S. cerevisiae* it was shown that acetylation of histone H3 is accomplished through the combined activity of two enzymes, Sas3 and Gcn5, the latter of which is a GNAT family histone acetyltransferase. Due to the overlapping pattern of acetylation via two enzymes, deletion of individual genes has a negligible effect, instead lack of both genes is known to cause synthetic lethality in yeast. Our data show that the single deletion of *MoSAS3* alone resulted in significant growth and developmental defects. This observation prompted us to investigate if such interaction is also conserved in *M. oryzae*.

**Figure 5:**
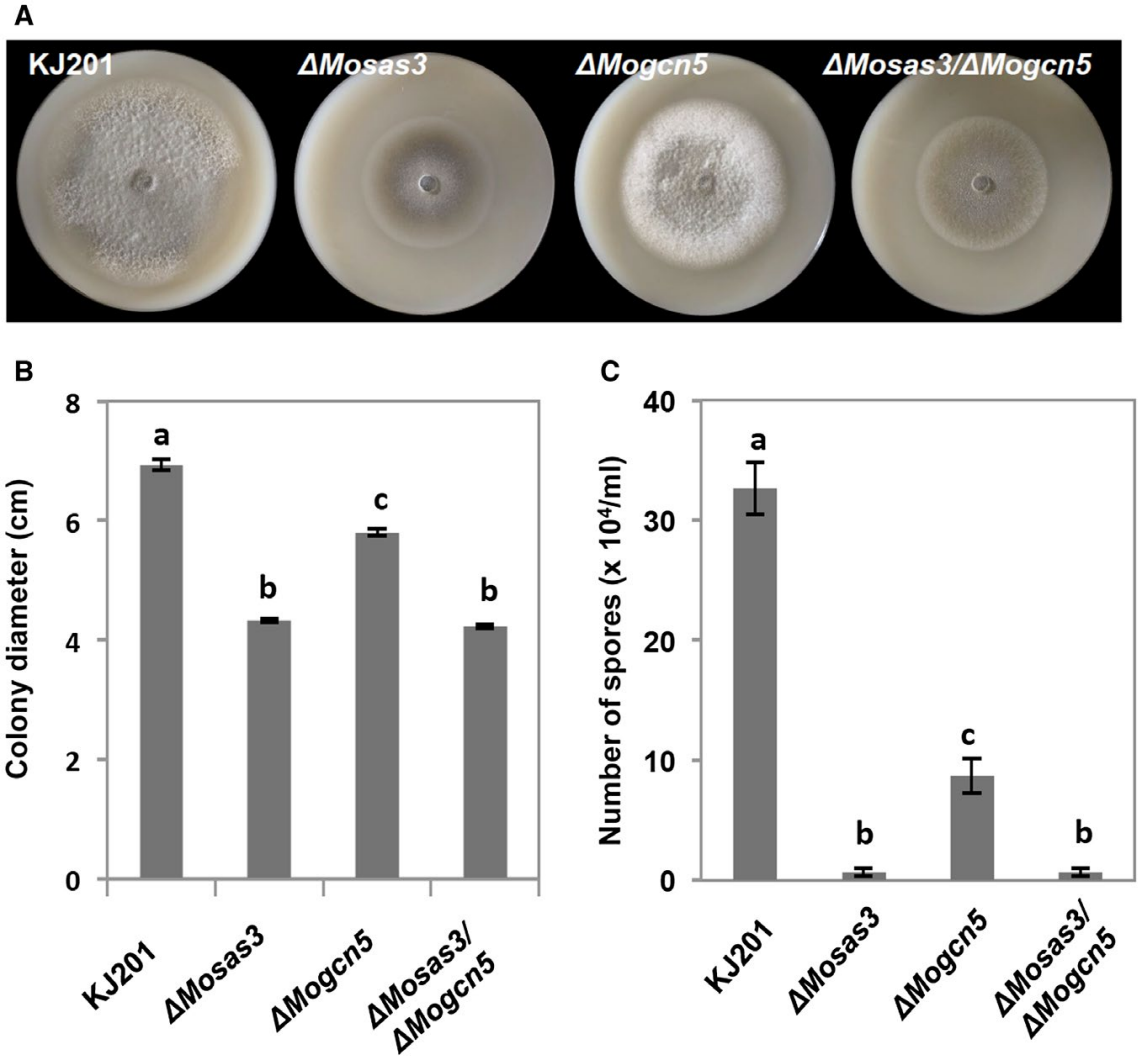
Test for genetic interaction between MoSAS3 and MoGCN5. (A) Comparison of growth on oatmeal agar plates for Δ*Mosas3*, Δ*Mogcn5* and the double deletion mutant (Δ*Mosas3/*Δ*Mogcn5*). (B) Growth of individual strains measured as colony diameter. (C) Asexual sporulation measured as the number of spores produced from individual strains. Different letters in bar plots indicate significantly different mean values (Tukey HSD test, *P* < 0.001).

Using dbHiMo, a web‐based database for histone‐modifying enzymes (http://hme.riceblast.snu.ac.kr/), we identified two genes (MGG_03677 and MGG_11716) encoding the putative Gcn5 orthologue in the *M. oryzae* genome and showed that two genes are probably the result of a relatively recent duplication event (Choi *et al*., 2015). A previous work investigating the role of GCN5 in light‐induced autophagy designated MGG_03677 and MGG_11716 as *MoGCN5* and *MoGCN5b*, respectively (Zhang *et al.*, 2017). Pairwise sequence alignment of MoGCN5 or MoGCN5b with *S. cerevisiae* Gcn5 using Needle (https://www.ebi.ac.uk/Tools/psa/emboss_needle/) indicated that MoGCN5 is more similar (56.6% identity and 71.3% similarity) to yeast Gcn5 than MoGCN5b (50.5% identity and 66.2% similarity). Moreover, deletion of *MoGCN5b* had no detectable effect, in contrast to deletion of *MoGCN5* rendering the fungus defective in growth, sporulation and pathogenicity (Figs 5 and S3, see Supporting Information). Sequence similarity, evolutionary history and phenotypes of deletion mutants suggested that MoGCN5 is a functional, bona fide histone acetyltransferase. Based on this, we tested if there is genetic interaction between *MoSAS3* and *MoGCN5* by generating a strain where both genes are deleted. Comparative analysis of different mutant strains showed that vegetative growth and asexual sporulation of the double deletion mutant (Δ*Mosas3/*Δ*Mogcn5*) are comparable to those of Δ*Mosas3*, indicating no genetic interaction of the kind that is found between the two genes in yeast (Fig. 5).

### Identifying *MoSAS3*‐dependent changes in gene expression of *M. oryzae*

To delve into the roles of *MoSAS3* in *M. oryzae*, we compared the transcriptome of Δ*Mosas3* with that of wild‐type strain by performing RNA‐seq on three biological replicates each for wild‐type and the mutant grown in complete media. Our RNA‐seq analysis showed that about 14.1% of genes (*n* = 1810) are differentially expressed in the mutant compared to the wild‐type, with statistically significant differences and a two‐fold expression change cut‐off. Among those differentially expressed genes (DEGs), about 44.9% (*n* = 813) and 54.1% (*n* = 997) were up‐regulated and down‐regulated in the mutant strain, respectively (Tables S1 and S2, see Supporting Information). Several genes that displayed transcriptional differences by RNA‐seq were validated through qRT‐PCR experiments (Fig. S4, see Supporting Information). Similar patterns were detected by RNA‐seq and qRT‐PCR (Spearman's rank‐order correlation = 0.87), indicating that we have accurately detected gene expression changes in our experiments.

### Altered expression profile of Δ*Mosas3* resembles that of Δ*Mortt109*

**Figure 6:**
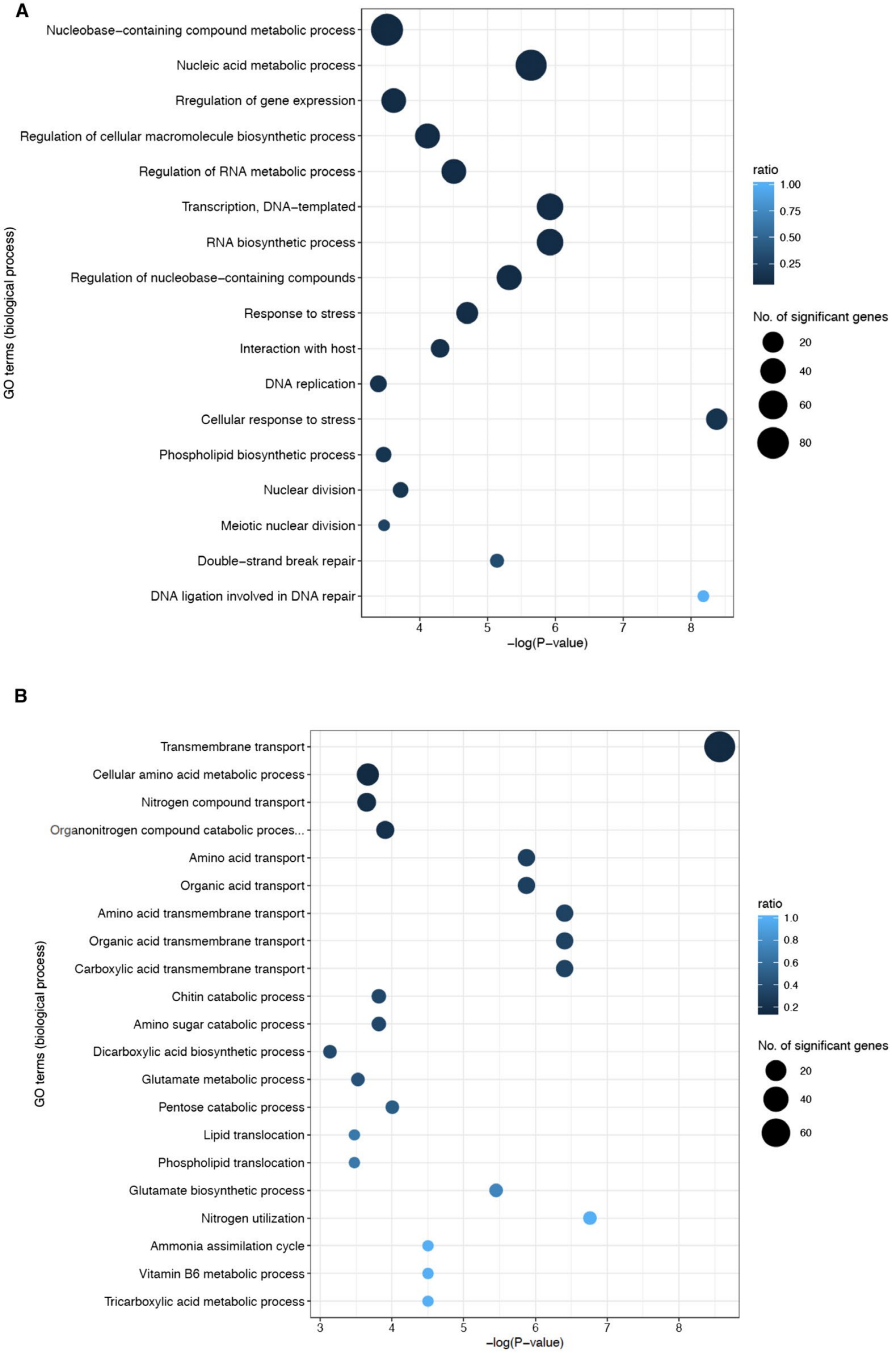
Summary of GO enrichment analysis (biological process) for selected terms. The whole GO enrichment analysis is available in the Supporting Information. GO terms that are enriched with up‐regulated and down‐regulated genes are shown in (A) and (B), respectively (Fisher's exact test, *P* < 0.01). The area of the circle represents the number of genes assigned to the particular GO term. The colour of the circle indicates the proportion of genes assigned to the GO term in our dataset among the total number of genes having that GO term in the genome.

To gain insight into transcriptomic changes associated with absence of *MoSAS3*, we performed gene ontology (GO) term analysis to quantify the enrichment of genes associated with biological processes. Our GO term analysis showed two general trends among DEGs: (i) up‐regulated genes were enrichment with functions including cellular response to stress, DNA metabolic process and DNA repair (Figs 6A and S5, see Supporting Information), and (ii) unlike the up‐regulated genes, the down‐regulated genes displayed enrichment with genes implicated in nutrient transport and diverse metabolic processes including nitrogen utilization, organic acid metabolism and vitamin metabolism (Figs 6B and S6, see Supporting Information). Recently, we demonstrated that a fungal‐specific histone acetyltransferase, MoRtt109, is required for proper regulation of the cell cycle as well as DNA damage repair and responses (Kwon *et al.*, 2018). We noticed that transcriptional output of the mutant bears a close resemblance to that of Δ*Mortt109.* Comparison of DEGs showed that there is highly significant overlap (*n* = 1006) between DEGs of Δ*Mosas3* (*n* = 1810) and Δ*Mortt109* (*n* = 1641), confirming our observation (hypergeometric test, *P* < 0.001) (Fig. S7, see Supporting Information). However, expression of *MoRtt109* itself was marginally up‐regulated (2.04‐fold change, *P* < 0.01) in Δ*Mosas3*, suggesting that similarity in expression profiles is created by different mechanisms in the two mutants.

### Deletion of *MoSAS3* alters expression of genes involved in cellular metabolism

In addition to DNA repair and damage response genes, some of the metabolic genes were up‐regulated in the mutant as well. One such example is trihydroxynaphthalene reductase (3HNR, MGG_07216), which is one of the catalytic enzymes for melanin biosynthesis. In the mutant, expression of 3HNR increased by nearly 45‐fold, and this might underlie the highly melanized mutant colonies in plate cultures, although expression of other melanin biosynthesis genes was not differentially expressed in submerged cultures (Fig. 2A). However, metabolic gene expression was globally down‐regulated in the mutant relative to the wild‐type. Detailed analysis of those metabolic genes revealed that many of the carbon metabolism genes involved in glycolysis and the citrate cycle were down‐regulated in the mutant (Tables S3 and S4, see Supporting Information). Furthermore, nitrogen utilization genes such as NADP‐specific glutamate dehydrogenase (MGG_08074), nitrate reductase (MGG_06062), nitrite reductase (MGG_00634) and nitrilase (MGG_03280) were down‐regulated (Table S5, see Supporting Information). Together with down‐regulation of genes involved in nutrient transport, our RNA‐seq data indicate that global down‐regulation of primary metabolism accounts for most, if not all, of the growth defects observed in Δ*Mosas3.*

Since transcription factors (TFs) are generally responsible for large‐scale changes in transcriptional output of the genome, we next investigated changes in the expression of genes encoding these regulatory proteins using the Fungal Transcription Factor Database (http://ftfd.snu.ac.kr/index.php?a=view) (Park *et al.*, 2008). Our analysis showed that deletion of *MoSAS3* does not equally influence expression of different families of TFs. Bromodomain and homeodomain TFs were up‐regulated in the mutant, while the Myb, Zn_2_Cys_6_, bHLH and bZIP families of TFs tend to be down‐regulated (Fig. S8, see Supporting Information). Interestingly, many of the knockout mutants for Zn_2_Cys_6_ and bZIP TFs were reported to have defects in nutrient utilization and/or hyphal growth (Lu *et al.*, 2014; Tang *et al.*, 2015). This suggests the possibility that at least some genes encoding these TFs are direct targets of *MoSAS3‐*mediated histone acetylation and that widespread changes in gene expression of the mutant may be a consequence of flow‐on effects resulting from a change in expression of the key genes that control transcription.

## Discussion

Sas3 and its homologues (MOZ/MORF, ENOK) are widely conserved across different kingdoms. The roles of MOZ/MORF, ENOK and yeast Sas3 have been well established in human diseases such as leukaemia (Rokudai *et al.*, 2009, 2013; Voss *et al.*, 2009), regulation of global acetylation levels in *Drosophila* (Scott *et al.*, 2001) and regulation of histone acetylation in yeast (Durant and Pugh, 2006; Reifsnyder *et al.*, 1996; Sterner and Berger, 2000; Takechi and Nakayama, 1999), respectively. However, compared to the GNAT family of HATs, study of the MYST family of HATs is considerably lacking. In this study, we investigated the roles of MoSAS3 as a MYST family HAT in fungal development and pathogenesis in order to bridge this knowledge gap in fungal pathogens.

We first examined cellular localization of MoSAS3 by expressing MoSAS3 protein fused to GFP at its C‐terminal. This showed that MoSAS3 is localized to fungal nuclei as expected for a HAT. However, a faint and diffuse fluorescence signal was also detected in the cytoplasm (Fig. 1B). It should be noted that despite heavy research focus on histones, post‐translation modifications (PTMs) are actually more widespread than previously imagined. Acetylation in particular was found to be a ubiquitous regulatory PTM, showing parallels to phosphorylation (Kouzarides, 2000). Considering that an increasing number of non‐histone targets of previously known HATs are being discovered, our data suggest that MoSAS3 is likely to have cytoplasmic targets as well. In an effort to understand the contribution of *MoSAS3* in fungal growth and development, we generated mutant strains where *MoSAS3* is deleted in the genome. Absence of *MoSAS3* in the fungal genome decreased the global acetylation level of H3K14 by approximately 40–50% (Figs 1C and S2, see Supporting Information). Together with conserved domain architecture and nuclear localization, this result strongly suggests that MoSAS3 is a bona fide histone acetyltransferase in *M. oryzae*.

Deletion of the *MoSAS3* gene significantly impaired vegetative mycelial growth compared to the wild‐type strain. The deletion mutant not only exhibited diminished radial growth, but also exhibited reduced thickness in the mycelial mat compared to the wild‐type strain, pointing towards the direct role of *MoSAS3* in regulating fungal vegetative growth. Our RNA‐seq analysis suggests that global down‐regulation of genes involved in primary metabolism is likely to underlie this growth defect. Under conditions where primary metabolism is repressed, we predicted two changes in the mutant transcriptome: an increase in recycle/salvage processes and a decrease in *de novo* protein production. Contrary to our expectation, however, genes involved in recycling of nitrogen resources such as exo‐peptidases and metallo‐peptidases were not up‐regulated. Autophagy is also an important mechanism for cellular recycling, but none of the autophagy genes were differentially regulated in the mutant. Furthermore, *de novo* protein synthesis involving tRNA synthetases and ribosome complexes, which is a highly energy demanding process, was not repressed in the mutant. Considering that RNA was extracted from mutant mycelia grown in complete medium, these results suggest that the mutant is probably able to sense the presence of nutrients in the extracellular milieu but it cannot properly express genes implicated in the efficient transport and use of those nutrients due to absence of MoSAS3‐mediated histone acetylation. It is noteworthy that among down‐regulated genes in the mutant are Zn_2_Cys_6_ and bZIP TFs, many of which are required for proper growth and asexual sporulation (Lu *et al.*, 2014; Tang *et al.*, 2015). In particular, systematic analysis of Zn_2_Cys_6_ TFs by Lu *et al*. showed that deletion of some Zn_2_Cys_6_ TFs leads to defects in growth, conidiation, germination and appressorium formation. Among 33 TFs that are down‐regulated in the *MoSAS3* deletion mutant, Zn_2_Cys_6_ TFs accounted for 42% (*n* = 13) in our RNA‐seq data. Such high enrichment of one type of TF suggests the possibility that MoSAS3‐mediated transcriptional regulation preferentially targets Zn_2_Cys_6_ TFs, which in turn control the transcription of a multitude of metabolic enzyme genes.

One unexpected observation in our RNA‐seq data was the close resemblance of gene expression profiles between Δ*Mosas3* and Δ*Mortt109.* In our previous study, we reported that MoRtt109 acetylates H3K56 and is involved in DNA repair and damage responses. In both mutants, DNA repair and damage response genes tended to be up‐regulated, while a diverse array of metabolic genes is down‐regulated. One plausible explanation for this is down‐regulation of *MoRtt109* in the Δ*Mosas3*. However, we can rule out this possibility since RNA‐seq data showed marginal up‐regulation of *MoRtt109* in the mutant (2.04‐fold, *P*  < 0.001), indicating that similarity in transcriptomic changes between two mutants is probably based on different mechanisms. Nevertheless, it is tempting to speculate that DNA repair/damage responses and cellular metabolism are linked at the epigenetic level.

Other than vegetative growth, we observed a sharp decline in the ability of the mutant to form conidia, which play a pivotal role as secondary inoculum in the disease cycle of the rice blast. Strikingly, conidia that are produced from the mutant were rarely able to germinate because they were metabolically inactive. To date, only a handful of genes that regulate conidiogenesis have been identified and characterized, and details of morphogenetic and metabolic processes associated with conidiogenesis are largely unknown. Considering misregulation of metabolism genes in the mutant mycelia, we conjecture that MoSAS3 might serve as an important regulator that orchestrates the metabolic changes required during conidiogenesis. Moreover, it appeared that such a defect in initial development has a detrimental effect on down‐stream processes in the mutant, impairing germination, appressorium formation and eventually pathogenesis. However, it should be noted that a number of different factors, including cytological malfunction of cellular organelles or defect in gene expression, may contribute to the inability of the mutant conidia to germinate. Our sheath assay showed that rice cells challenged with Δ*Mosas3* accumulate brown granules and/or show discolouration. Such a response is typically viewed as being associated with inability of the fungus to suppress plant immune responses and is reminiscent of the Δ*Modes1* phenotype (Chi *et al.*, 2009), although expression of *MoDES1* was not altered in Δ*Mosas3* compared to the wild‐type. However, it cannot be ruled out that *MoDES1* expression is under faulty regulation during the early stage of plant infection. It is noteworthy that a large proportion of genes encoding putative secreted proteins are misregulated in the mutant (Fig. S9, see Supporting Information). Collectively, these observations suggest that many fungal genes involved in interaction with host plants seem to be affected by *MoSAS3*.

Given that deletion of *Sas3* in yeast has little impact on phenotypes (Rosaleny *et al.*, 2007), it is interesting to see large effects of *MoSAS3* deletion on development and pathogenicity in *M. oryzae*. Yeast Sas3 has been shown to function in association with GNAT family HAT, Gcn5 (Rosaleny *et al.*, 2007). In contrast to deletion of the yeast gene pair Gcn5 and Sas3, showing synthetic lethality, deletion of *MoGCN5* in Δ*Mosas3* background did not change the phenotypes of Δ*Mosas3*, suggesting different integration of two evolutionarily conserved components into the epigenetic regulatory system between *S. cerevisiae* and *M. oryzae.* We propose that two scenarios, which are not mutually exclusive, can explain such a difference between the two species. First, most if not all MoGCN5 targets could be a subset of MoSAS3 targets. Second, MoSAS3 can have cytoplasmic targets, as evidenced by our MoSAS3 protein localization data, unlike its yeast counterpart. It is of note that MoGCN5 has also been shown to have cytoplasmic targets, one of which is an autophagy protein, MoATG7 (MGG07297) (Zhang *et al.*, 2017). This is in stark contrast to *S. cerevisiae*, where Esa1, which is a part of the NuA4 complex that is responsible for global H4 acetylation, is the only yeast HAT so far known to have non‐histone substrates (Sapountzi and Cote, 2011). Taken together, these data suggest that there is a degree of functional variation and target protein specificity within each group, and that there is a caveat when generalizing and newly predicting the biological function of acetyltransferases in other species based on previous studies in model organisms.

In summary, our data indicate that MoSAS3 is a conserved epigenetic component responsible for acetylation of H3K14. Although MoSAS3 is likely to have non‐histone substrates as well, such HAT activity seems to be important for growth and pre‐penetration development in *M. oryzae*. Transcriptome data provide a novel insight into the profound implications of MoSAS3 on DNA damage responses/repair and regulation of cellular metabolisms. Further elucidation of target genes and potential non‐histone substrates would shed light on species‐specific quirks and surprises, and subsequent development of a novel control strategy for fungal diseases of plants.

## Experimental Procedures

### Fungal isolates and culture conditions

The wild‐type strain *M. oryzae* KJ201 was procured from the Center for Fungal Genetic Resources (CFGR), South Korea. All strains, including mutants, were grown on oatmeal agar plates (OMA) [5% oatmeal (w/v) and 2% agar (w/v)] at 25 °C under constant white light to promote conidiation. Three‐day‐old vegetative hyphae harvested from the complete medium [CM, 0.6% yeast extract (w/v), 0.6% casamino acid (w/v) and 1% sucrose (w/v); 25 °C with 120 rpm shaking] were used for RNA and DNA extraction. Selection of hygromycin‐resistant transformants was carried out using TB3 agar plates [0.3% yeast extract (w/v), 0.3% casamino acid (w/v), 1% glucose (w/v), 20% sucrose (w/v) and 0.8% agar powder (w/v)] supplemented with 200 ppm hygromycin B.

### Test of growth, conidiation and appressorium formation

For measurement of radial mycelial growth rate, conidiation, germination and appressorium formation, strains were cultured on either V8 agar or OMA plates in three replicates for 9 days at 25 °C under continuous light conditions. Conidia were harvested from 9‐day‐old mycelia in 5 mL of sterile water. Conidiation was determined by counting the number of conidia using a haemocytometer under the light microscope. Next, the conidial suspension was adjusted to a concentration of 5 × 10^4^ conidia/mL. Forty microlitres of the conidial suspension was dropped onto plastic coverslips with three replicates and incubated in a humidity box at 25 °C. After 4 h of incubation, the frequency of conidial germination was determined by counting at least 100 conidia per replicate under the bright field microscope. Eight hours after incubation, the frequency of appressorium formation was determined by counting number of conidia that formed appressoria among germinated conidia (at least 100 total conidia in each replicate) under the light microscope. All the assays were performed with three replicates in three independent experiments. Slides were examined under a Leica DM2500 light microscope and imaged with a Leica DFC7000 T digital camera using Leica Application Suite v4.

### Pathogenicity assay

For pathogenicity assay, we either sprayed spore suspensions (*c*.10^5^/mL) directly onto the rice seedlings or injected into the leaves of rice seedlings (*Oryza sativa* ‘Nakdongbyeo’) of three‐ to four‐leaf stages through wound sites. The inoculated plants were placed in a humidity box for 24 h under dark conditions at 25 °C, then transferred to an incubator at 25 °C with a 16‐h photoperiod with fluorescent lights. Disease lesions were assessed and photographed at 7 days post‐inoculation. Plants grown in three pots were used for pathogenicity assay of individual strains in each of three independent experiments.

### Targeted gene deletion and complementation

Knockout constructs were generated using the strategy of double‐joint PCR (Yu *et al.*, 2004). For this, about 1.5 kb of flanking sequences of each gene were amplified and fused on either side of the hygromycin resistance gene (HPH) cassette. Next, the knockout constructs were transformed into the wild‐type protoplasts to generate transformants. Candidate transformants were screened using a PCR‐assisted screening method using specific primer pairs for the genes. Positive candidate knockout mutants were confirmed by Southern blot analysis using one of the flanking sequences as a probe. Southern blot DNA hybridization was performed with the selected transformants to ensure correct gene replacement events and absence of ectopic integration. Genomic DNA was digested with *Eco*R1, and blots were probed with 1‐kb 5′‐flanking or 3′‐flanking sequences (Fig. S1). Southern blot DNA hybridization was performed using a standard method. Complementation experiments on knockout mutants were carried out by amplifying the target gene and flanking sequences (~1 kb on each side) from the wild‐type and introducing the resulting PCR products along with pII99 vector harbouring the geneticin resistance gene marker into the mutant protoplasts. Geneticin‐resistant colonies were selected and subsequently screened by PCR for the presence of the target gene.

### Western blot analysis and histone acetyltransferase assay for H3K14 acetylation

Total protein extracted from mycelia was separated by 12% SDS‐PAGE. Blots were probed with histone H3 acetyl‐lysine K14 antibody (1:2000, Cat. No. 39599, Active Motif, CA, USA) and H3 antibody as loading control (1:2000, Cat. No. 39164, Active Motif, CA, USA). Pierce Fast Western Blot Kit, ECL Substrate (Pierce Biotechnology, Rockford, IL, USA) was used and signals were detected on X‐ray films. Independent measurement of global H3K14 acetylation levels was performed using EpiQuik Global Acetyl Histone H3‐K14 Quantification Kit (colourimetric) according to the manufacturer's instruction (Epigentek, Farmingdale, NY, USA).

### RNA sequencing and real‐time PCR analysis

Three‐day‐old mycelia harvested from the complete medium were used for total RNA extraction using an Easy‐spin total RNA extraction kit (iNtRON Biotechnology, Seoul, Korea) for all the strains. In RNA‐seq experiment, paired‐end sequencing of mRNAs from three biological replicates was carried out on the Illumina platform. TopHat was used to map the reads to the annotated *M. oryzae* transcripts, followed by analysis of transcript abundance and differential expression between the strains using Cufflinks. Expression levels were estimated as the number of fragments per kilobase per million reads (FPKM). Differentially expressed genes between the wild‐type and mutant strain were detected at the 0.01 significance level and with two‐fold change criteria. The FDR was used to maintain the significance level at 0.01. Fold changes of expression levels were calculated after adding one to each FPKM value to avoid division by zero. Gene ontology was carried out using the topGO package in R programming (Alexa and Rahnenfuhrer, 2019). Enrichment of genes in particular GO terms was analysed using Fisher's exact test (*P* < 0.01). For qRT‐PCR, 1 μg of total RNA was reverse transcribed into first‐strand cDNA with ImProm‐II Reverse Transcription System (Promega, Madison, WI, USA). Real‐time PCR was performed in a 20 μL volume containing 2 μL of cDNA (25 ng of input RNA), 1 μM of primer mix (forward and reverse) and 10 μL of 2 × Power SYBR Green PCR Master Mix (Applied Biosystems, Warrington, UK). Reactions were run on the Applied Biosystems 7500 Real Time PCR System (Applied Biosystems, Foster City, CA, USA) for 40 cycles of 15 s at 95 °C, 30 s at 60 °C and 30 s at 72 °C with three technical replicates. Resulting values of mean threshold cycles (Ct) were normalized as previously described (Livak and Schmittgen, 2001). The results of real‐time PCR were confirmed through three independent experiments.

## Supporting information


**Fig. S1** Targeted gene replacement of *MoSAS3* (here shown as MoHAT10). Flanking sequences of *MoSAS3 *were amplified and fused to hygromycin cassette using double‐joint PCR (A). This PCR product was directly used for transformation of wild‐type protoplasts. Resulting transformants were screened by PCR (B) and correct gene replacement event was confirmed by Southern blot analysis (C). Location of primers used in PCR‐based screening (B) are designated in (A) as blue arrows. Primer pairs are supposed to give *c*.3 kb products only in the deletion mutant, and bands in the lower position in the gel appear to be non‐specific products.

**Fig. S2** Histone acetyltransferase activity of MoSAS3 and MoGCN5. (A) Western blot analysis of global H3K14 acetylation level in the Δ*Mosas3* strain compared to wild‐type (KJ201) and complementation strain (*MoSAS3* *c*). Total protein extracted was separated on a 15% polyacrylamide gel and probed with antibodies against H3K14ac. (B) Independent measurement of H3K14 acetylation levels in Δ*Mosas3* and Δ*Mogcn5 *compared to the wild‐type (EpiQuik Global Acetyl Histone H3‐K14 Quantification Kit, Epigentek).

**Fig. S3 **Phenotypes of Δ*Mogcn5*. Germination and appressorium formation of Δ*Mogcn5* (A, B and C). Reduced pathogenicity of Δ*Mogcn5* on host plants (D and E). Asterisks indicate statistically significant differences (*P* < 0.001, Tukey HSD test).

**Fig. S4** Validation of RNA‐seq data via qRT‐PCR. Twelve (six up‐ and six down‐regulated) differentially expressed genes in our RNA‐seq data were randomly selected and their transcript abundance in the mutant relative to the wild‐type was examined using qRT‐PCR. Mean Ct values were obtained from three technical replicates. The consistent pattern of expression observed here was confirmed via two independent experiments. Correlation between qRT‐PCR and RNA‐seq data was calculated using Spearman's rank‐order in R programming language.

**Fig. S5** Summary of GO enrichment analysis (biological process). GO terms that are enriched with up‐regulated genes are shown (Fisher's exact test, *P* < 0.01). The area of the circle represents the number of genes assigned to the particular GO term. The colour of the circle indicates the proportion of genes assigned to the GO term in our data set among the total number of genes having that GO term in the genome.

**Fig. S6** Summary of GO enrichment analysis (biological process). GO terms that are enriched with down‐regulated genes are shown (Fisher's exact test, *P* < 0.01). The area of the circle represents the number of genes assigned to the particular GO term. The colour of the circle indicates the proportion of genes assigned to the GO term in our data set among the total number of genes having that GO term in the genome.

**Fig. S7** Venn diagram summarizing the overlap of differentially expressed genes between Δ*Mosas3 *and Δ*Mortt109.*

**Fig. S8** Genes encoding transcription factors (TF) that are differentially expressed in Δ*Mosas3*. Percentage (*y*‐axis) of up‐ or down‐regulated (red and green bars, respectively) TF genes are shown relative to the total number of differentially expressed TFs (blue bars) across different families of TFs (*x*‐axis).

**Fig. S9** Number of differentially expressed genes among putative secreted protein‐coding genes. A whole list of putative secreted proteins was retrieved from the Fungal Secretome Database (http://fsd.snu.ac.kr/). SP and SP3 indicate secreted proteins predicted by SignalP and three other signal peptide prediction programs, respectively.

**Table S1** List of up‐regulated genes in Δ*Mosas3.*

**Table S2** List of down‐regulated genes in Δ*Mosas3.*

**Table S3** RNA‐seq analysis on genes involved in glycolysis.

**Table S4** RNA‐seq analysis on genes involved in TCA cycle.

**Table S5** Changes in expression of nitrogen metabolism genes in *Mosas3* relative to the wild‐type.
