# Supplementary material for: A MYST family histone acetyltransferase, MoSAS3, is required for development and pathogenicity in the rice blast fungus
Source: Mol Plant Pathol. 2019 Jul 30;20(11):1491–505. doi: 10.1111/mpp.12856 (PMC6804344; doi:10.1111/mpp.12856)
Supplement: Supplementary file 1 — Fig. S1 Targeted gene replacement of MoSAS3 (here shown as MoHAT10). Flanking sequences of MoSAS3 were amplified and fused to hygromycin cassette using double‐joint PCR (A). This PCR product was directly used for transformation of wild‐type protoplasts. Resulting transformants were screened by PCR (B) and correct gene replacement event was confirmed by Southern blot analysis (C). Location of primers used in PCR‐based screening (B) are designated in (A) as blue arrows. Primer pairs are supposed to give c.3 kb products only in the deletion mutant, and bands in the lower position in the gel appear to be non‐specific products. [file MPP-20-1491-s001.pdf]

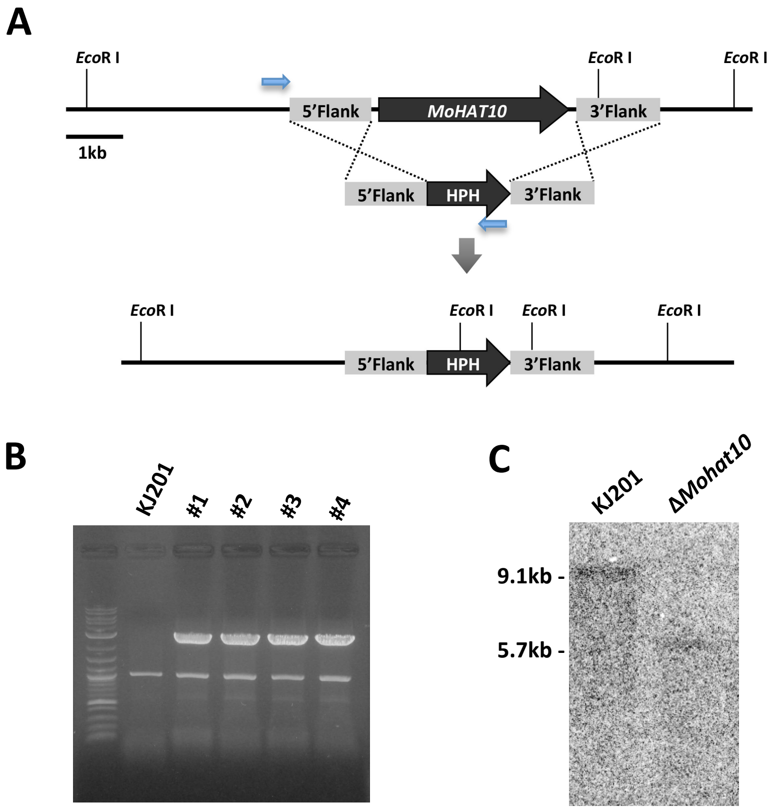

Fig. S1 Targeted gene replacement of *MoSAS3* (here shown as MoHAT10). Flanking sequences of *MoSAS3* were amplified and fused to hygromycin cassette using double-joint PCR (A). This PCR product was directly used for transformation of wild-type protoplasts. Resulting transformants were screened by PCR (B) and correct gene replacement event was confirmed by Southern blot analysis (C). Location of primers used in PCR-based screening (B) are designated in (A) as blue arrows. Primer pairs are supposed to give ~ 3kb products only in the deletion mutant, and bands in lower position in the gel appear to be non-specific products.
