# Supplementary material for: A MYST family histone acetyltransferase, MoSAS3, is required for development and pathogenicity in the rice blast fungus
Source: Mol Plant Pathol. 2019 Jul 30;20(11):1491–505. doi: 10.1111/mpp.12856 (PMC6804344; doi:10.1111/mpp.12856)
Supplement: Supplementary file 2 — Fig. S2 Histone acetyltransferase activity of MoSAS3 and MoGCN5. (A) Western blot analysis of global H3K14 acetylation level in the ΔMosas3 strain compared to wild‐type (KJ201) and complementation strain (MoSAS3 c). Total protein extracted was separated on a 15% polyacrylamide gel and probed with antibodies against H3K14ac. (B) Independent measurement of H3K14 acetylation levels in ΔMosas3 and ΔMogcn5 compared to the wild‐type (EpiQuik Global Acetyl Histone H3‐K14 Quantification Kit, Epigentek). [file MPP-20-1491-s002.pdf]

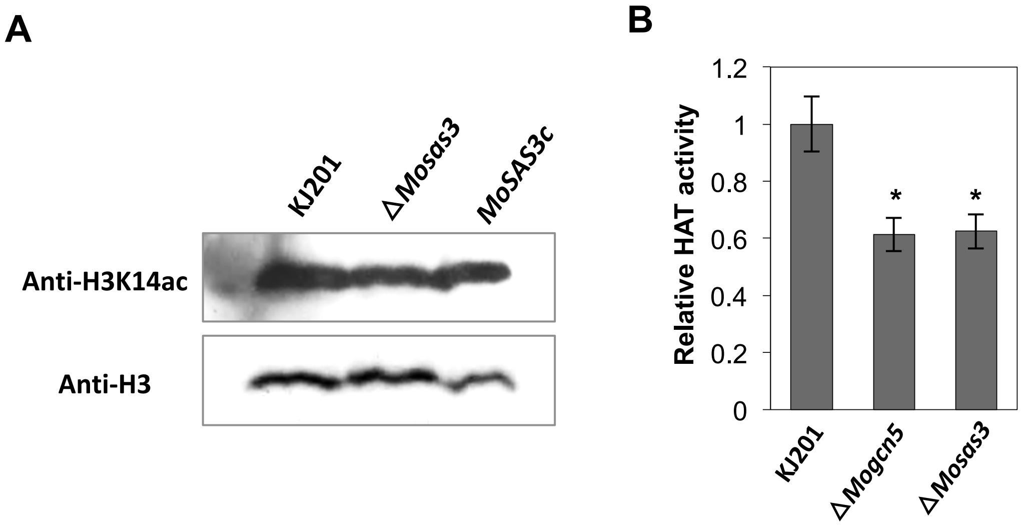

Fig. S2 Histone acetyltransferase activity of MoSAS3 and MoGCN5. (A) Western blot analysis of global H3K14 acetylation level in the *ΔMosas3* strain compared to wild type (KJ201) and complementation strain (*MoSAS3*c). Total protein extracted was separated on a 15% polyacrylamide gel and probed with antibodies against H3K14ac. (B) Independent measurement of H3K14 acetylation levels in Δ*Mosas3* and Δ*Mogcn5,* compared to the wild-type (EpiQuik^{TM} Global Acetyl Histone H3-K14 Quantification Kit, Epigentek). Asterisk indicates statistically signficant difference (TukeyHSD, $P < 0.001$).
