# Supplementary material for: A MYST family histone acetyltransferase, MoSAS3, is required for development and pathogenicity in the rice blast fungus
Source: Mol Plant Pathol. 2019 Jul 30;20(11):1491–505. doi: 10.1111/mpp.12856 (PMC6804344; doi:10.1111/mpp.12856)
Supplement: Supplementary file 3 — Fig. S3 Phenotypes of ΔMogcn5. Germination and appressorium formation of ΔMogcn5 (A, B and C). Reduced pathogenicity of ΔMogcn5 on host plants (D and E). Asterisks indicate statistically significant differences (P < 0.001, Tukey HSD test). [file MPP-20-1491-s003.pdf]

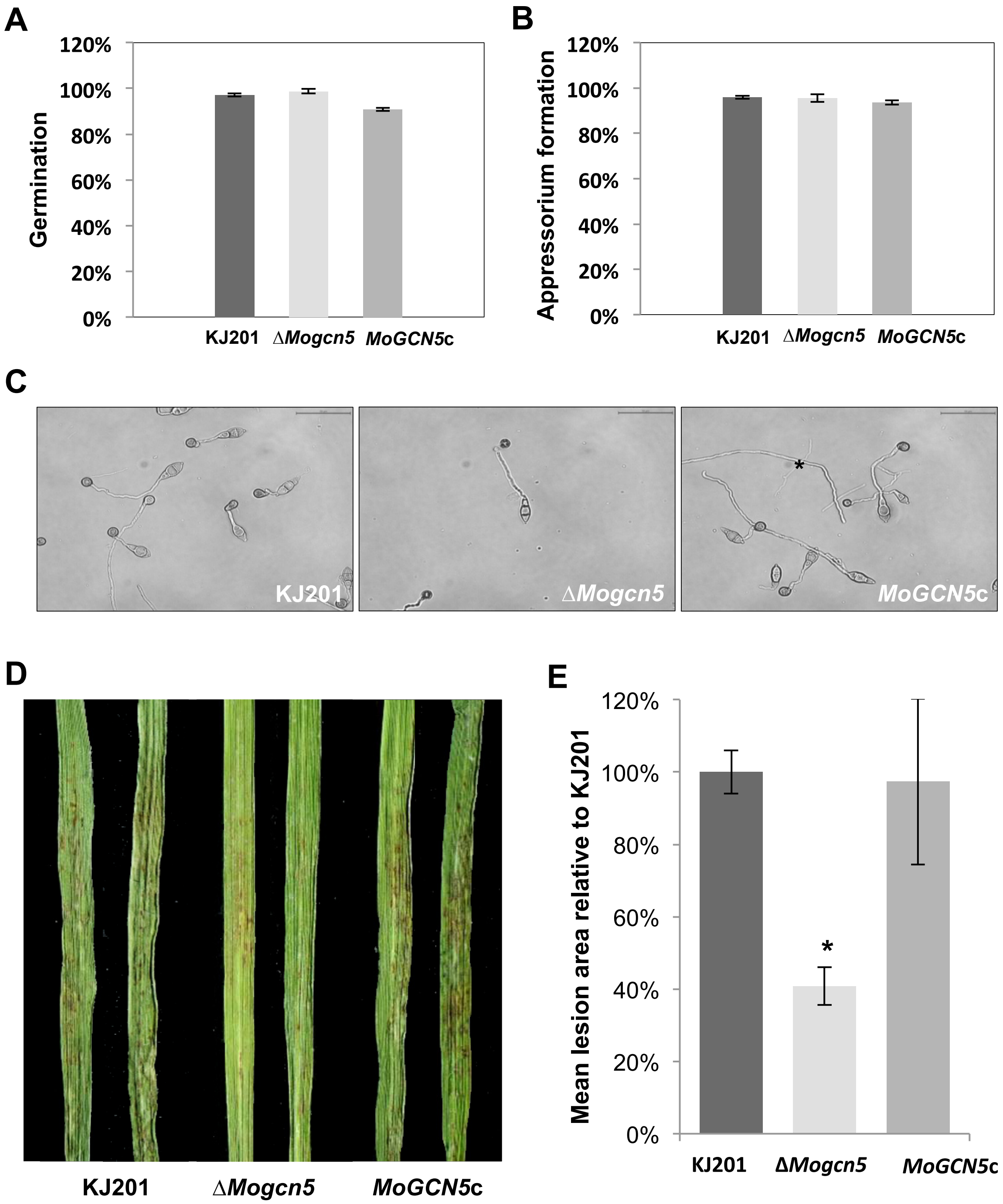

Fig. S3 Phenotypes of Δ*Mogcn5*. Germination and appressorium formation of Δ*Mogcn5* (A, B, and C). Reduced pathogenicity of Δ*Mogcn5* on host plants (D and E). Asterisks indicate statistically significant differences ($P <$ 0.001, TukeyHSD test).
