# Supplementary material for: A MYST family histone acetyltransferase, MoSAS3, is required for development and pathogenicity in the rice blast fungus
Source: Mol Plant Pathol. 2019 Jul 30;20(11):1491–505. doi: 10.1111/mpp.12856 (PMC6804344; doi:10.1111/mpp.12856)
Supplement: Supplementary file 4 — Fig. S4 Validation of RNA‐seq data via qRT‐PCR. Twelve (six up‐ and six down‐regulated) differentially expressed genes in our RNA‐seq data were randomly selected and their transcript abundance in the mutant relative to the wild‐type was examined using qRT‐PCR. Mean Ct values were obtained from three technical replicates. The consistent pattern of expression observed here was confirmed via two independent experiments. Correlation between qRT‐PCR and RNA‐seq data was calculated using Spearman's rank‐order in R programming language. [file MPP-20-1491-s004.pdf]

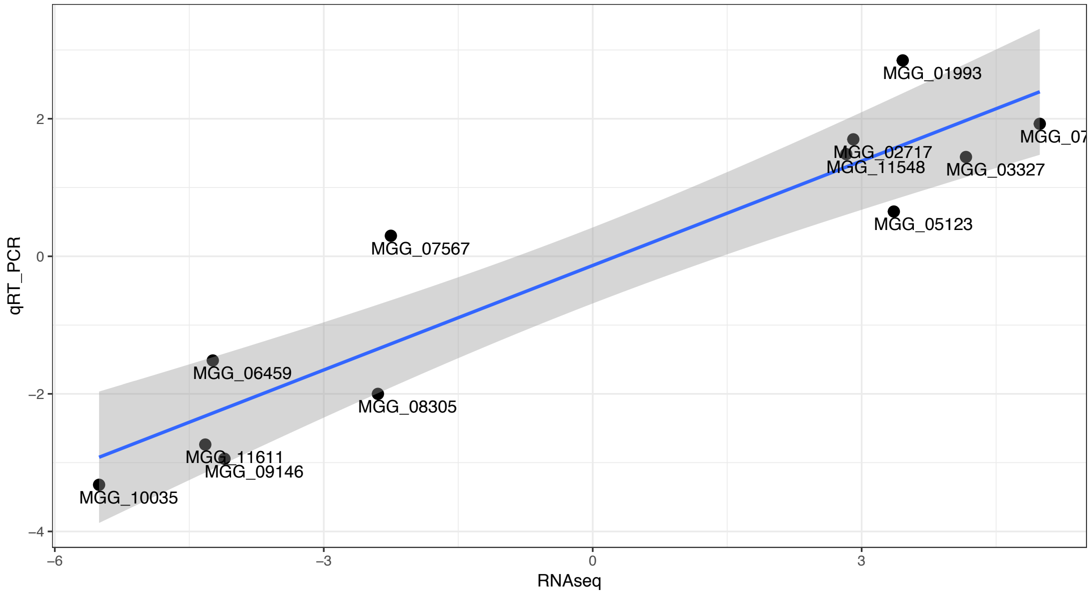

Fig. S4 Validation of RNA-seq data via qRT-PCR. Twelve (6 up- and 6 down-regulated genes) differentially expressed genes in our RNA-seq data were randomly selected and their transcript abundance in the mutant relative to the wild-type was examined using qRT-PCR.
