# Supplementary material for: A MYST family histone acetyltransferase, MoSAS3, is required for development and pathogenicity in the rice blast fungus
Source: Mol Plant Pathol. 2019 Jul 30;20(11):1491–505. doi: 10.1111/mpp.12856 (PMC6804344; doi:10.1111/mpp.12856)
Supplement: Supplementary file 5 — Fig. S5 Summary of GO enrichment analysis (biological process). GO terms that are enriched with up‐regulated genes are shown (Fisher's exact test, P < 0.01). The area of the circle represents the number of genes assigned to the particular GO term. The colour of the circle indicates the proportion of genes assigned to the GO term in our data set among the total number of genes having that GO term in the genome. [file MPP-20-1491-s005.pdf]

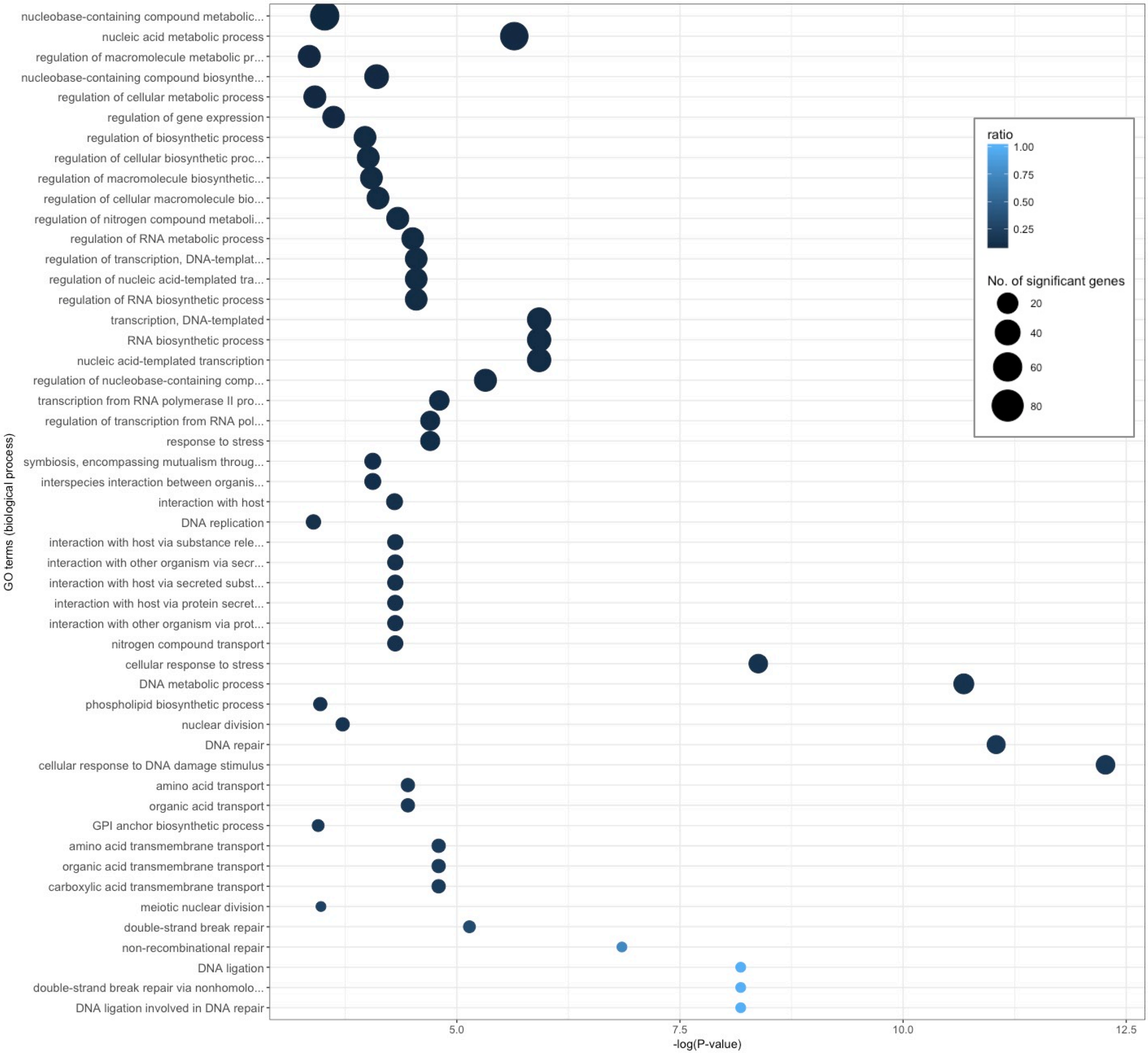

Fig. S5 Validation of RNA-seq data via qRT-PCR. Twelve (6 up- and 6 down-regulated genes) differentially expressed genes in our RNA-seq data were randomly selected and their transcript abundance in the mutant relative to the wild-type was examined using qRT-PCR.
