# Supplementary material for: A MYST family histone acetyltransferase, MoSAS3, is required for development and pathogenicity in the rice blast fungus
Source: Mol Plant Pathol. 2019 Jul 30;20(11):1491–505. doi: 10.1111/mpp.12856 (PMC6804344; doi:10.1111/mpp.12856)
Supplement: Supplementary file 7 — Fig. S7 Venn diagram summarizing the overlap of differentially expressed genes between ΔMosas3 and ΔMortt109. [file MPP-20-1491-s007.pdf]

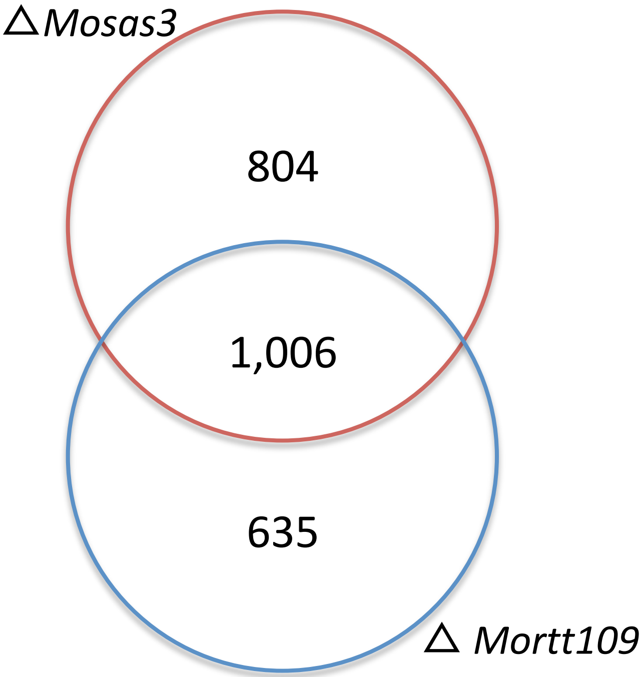

Fig. S7 Venn diagram summarizing overlap of differentially expressed genes between Δ*Mosas3* and Δ*Mortt109*.
