# Supplementary material for: A MYST family histone acetyltransferase, MoSAS3, is required for development and pathogenicity in the rice blast fungus
Source: Mol Plant Pathol. 2019 Jul 30;20(11):1491–505. doi: 10.1111/mpp.12856 (PMC6804344; doi:10.1111/mpp.12856)
Supplement: Supplementary file 8 — Fig. S8 Genes encoding transcription factors (TF) that are differentially expressed in ΔMosas3. Percentage (y‐axis) of up‐ or down‐regulated (red and green bars, respectively) TF genes are shown relative to the total number of differentially expressed TFs (blue bars) across different families of TFs (x‐axis). [file MPP-20-1491-s008.pdf]

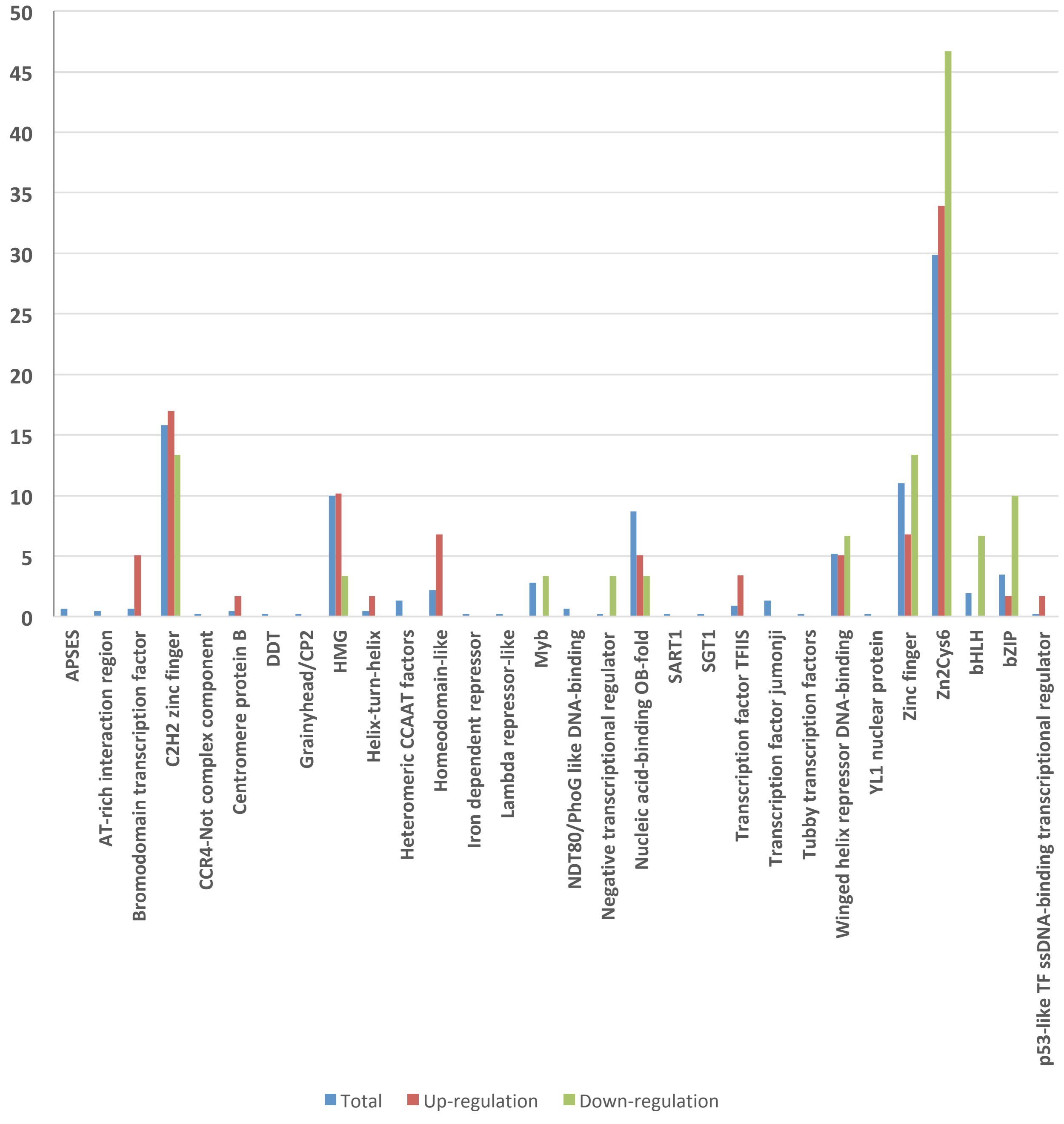

Fig. S8 Genes encoding transcription factors (TF) that are differentially expressed in ΔMosas3. Percentage (y-axis) of up- or down-regulated (red and green bars, respectively) TF genes are shown relative to the total number of differentially expressed TFs (blue bars) across different families of TFs (x-axis).
