# Supplementary material for: A MYST family histone acetyltransferase, MoSAS3, is required for development and pathogenicity in the rice blast fungus
Source: Mol Plant Pathol. 2019 Jul 30;20(11):1491–505. doi: 10.1111/mpp.12856 (PMC6804344; doi:10.1111/mpp.12856)
Supplement: Supplementary file 9 — Fig. S9 Number of differentially expressed genes among putative secreted protein‐coding genes. A whole list of putative secreted proteins was retrieved from the Fungal Secretome Database (http://fsd.snu.ac.kr/). SP and SP3 indicate secreted proteins predicted by SignalP and three other signal peptide prediction programs, respectively. [file MPP-20-1491-s009.pdf]

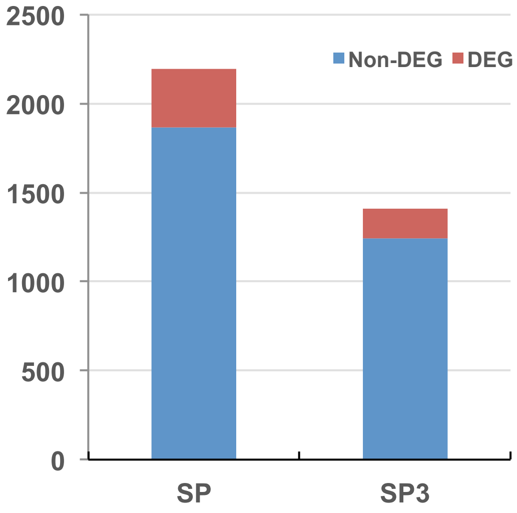

Fig. S9 Number of differentially expressed genes among putative secreted protein-coding genes. A whole list of putative secreted proteins were retrieved from Fungal Secretome Database (http://fsd.snu.ac.kr/). SP and SP3 indicate secreted proteins predicted by SignalP and three other signal peptide prediction programs, respectively.
