# Supplementary material for: A MYST family histone acetyltransferase, MoSAS3, is required for development and pathogenicity in the rice blast fungus
Source: Mol Plant Pathol. 2019 Jul 30;20(11):1491–505. doi: 10.1111/mpp.12856 (PMC6804344; doi:10.1111/mpp.12856)
Supplement: Supplementary file 14 — Table S5 Changes in expression of nitrogen metabolism genes in Mosas3 relative to the wild‐type. [file MPP-20-1491-s014.docx]

Table S5. Changes in expression of nitrogen metabolism genes in Δ*Mosas3* relative to the wild-type (down-regulation in green and up-regulation in red)

| **Gene** | **Annotation** | **Fold-change** |
| --- | --- | --- |
| MGG_17079 | formamidase | 0.57 |
| MGG_13793 | nitrate transporter | 0.64 |
| MGG_06062 | nitrate reductase | 0.31 |
| MGG_00634 | nitrite reductase | 0.32 |
| MGG_07261 | 2-nitropropane dioxygenase | 0.44 |
| MGG_02593 | 2-nitropropane dioxygenase | 0.86 |
| MGG_03280 | nitrilase 2 | 0.42 |
| MGG_05247 | NAD-specific glutamate dehydrogenase | 0.67 |
| MGG_08074 | NADP-specific glutamate dehydrogenase | 0.18 |
| MGG_14279 | glutamine synthetase | 1.39 |
| MGG_02538 | glutamine synthetase | 1 |
| MGG_06888 | glutamine synthetase | 0.52 |
| MGG_07187 | glutamate synthase | 0.38 |
| MGG_04611 | carbonic anhydrase | 2.75 |
| MGG_18017 | hypothetical protein | 1.43 |
| MGG_09234 | carbonate dehydratase | 1.09 |
